# Early-Life Iron Deficiency Reduces Brain Iron Content and Alters Brain Tissue Composition Despite Iron Repletion: A Neuroimaging Assessment

**DOI:** 10.3390/nu10020135

**Published:** 2018-01-27

**Authors:** Austin T. Mudd, Joanne E. Fil, Laura C. Knight, Fan Lam, Zhi-Pei Liang, Ryan N. Dilger

**Affiliations:** 1 Piglet Nutrition & Cognition Laboratory, University of Illinois Urbana-Champaign, Urbana, IL 61801, USA; jfil2@illinois.edu (J.E.F.); knight24@illinois.edu (L.C.K.); 2 Neuroscience Program, University of Illinois Urbana-Champaign, Urbana, IL 61801, USA; 3 Division of Nutrition Sciences, University of Illinois Urbana-Champaign, Urbana, IL 61801, USA; 4 Beckman Institute for Advanced Science & Technology, University of Illinois Urbana-Champaign, Urbana, IL 61801, USA; fanlam1@illinois.edu (F.L.); z-liang@illinois.edu (Z.-P.L.); 5 Department of Electrical & Computer Engineering, University of Illinois Urbana-Champaign, Urbana, IL 61801, USA; 6 Department of Animal Sciences, University of Illinois Urbana-Champaign, Urbana, IL 61801, USA

**Keywords:** neurodevelopment, iron deficiency, pig, iron repletion, myelination, pediatric nutrition, brain iron

## Abstract

Early-life iron deficiency has lifelong influences on brain structure and cognitive function, however characterization of these changes often requires invasive techniques. There is a need for non-invasive assessment of early-life iron deficiency with potential to translate findings to the human clinical setting. In this study, 28 male pigs were provided either a control diet (CONT; *n* = 14; 23.5 mg Fe/L milk replacer) or an iron-deficient diet (ID; *n* = 14; 1.56 mg Fe/L milk replacer) for phase 1 of the study, from postnatal day (PND) 2 until 32. Twenty pigs (*n* = 10/diet from phase 1 were used in phase 2 of the study from PND 33 to 61, where all pigs were provided a common iron-sufficient diet, regardless of their phase 1 dietary iron status. All pigs were subjected to magnetic resonance imaging at PND 32 and again at PND 61, and quantitative susceptibility mapping was used to assess brain iron content at both imaging time-points. Data collected on PND 61 were analyzed using voxel-based morphometry and tract-based spatial statistics to determine tissue concentration difference and white matter tract integrity, respectively. Quantitative susceptibility mapping outcomes indicated reduced iron content in the pons, medulla, cerebellum, left cortex, and left hippocampus of ID pigs compared with CONT pigs, regardless of imaging time-point. In contrast, iron contents were increased in the olfactory bulbs of ID pigs compared with CONT pigs. Voxel-based morphometric analysis indicated increased grey and white matter concentrations in CONT pigs compared with ID pigs that were evident at PND 61. Differences in tissue concentrations were predominately located in cortical tissue as well as the cerebellum, thalamus, caudate, internal capsule, and hippocampi. Tract-based spatial statistics indicated increased fractional anisotropy values along subcortical white matter tracts in CONT pigs compared with ID pigs that were evident on PND 61. All described differences were significant at *p* ≤ 0.05. Results from this study indicate that neuroimaging can sensitively detect structural and physiological changes due to early-life iron deficiency, including grey and white matter volumes, iron contents, as well as reduced subcortical white matter integrity, despite a subsequent period of dietary iron repletion.

## 1. Introduction

Iron deficiency is the most common micronutrient deficiency worldwide [1,2] and a deficiency during the perinatal period has lifelong implications. Infants are at an increased risk for iron deficiency [3] and the developing brain is highly vulnerable to alterations in iron status [4,5,6,7]. Research in humans has shown that iron deficiency early in life results in delayed motor development by ten months of age [8], delayed cognitive processing by ten years of age [9], altered recognition memory and executive functions at 19 years of age [10], and poorer emotional health in the mid-twenties [11]. It is clear that early-life iron deficiency has lasting effects on cognitive performance, yet it remains to be elucidated what structural differences in brain development might underlie these persistent cognitive changes. Some studies in humans have used other non-invasive techniques such as evoked potential recordings [12], electrophysiological recording and processing [9] and electroencephalography [13] to explain structural alterations in brain development related to iron deficiency. While these methods may potentially explain a mechanism for altered brain development, more sensitive assessments are needed to non-invasively characterize the brain regions influenced by iron deficiency.

The presence of iron in the brain is necessary for proper vascular development [14], myelination [15], neurotransmitter synthesis [16,17], and neuron morphology [18,19]. To date, many of the mechanisms for iron’s involvement in brain development have been determined using invasive techniques in animal models. While necessary and informative, these methods are not feasible in clinical populations, thus there is a need to characterize similar findings using non-invasive techniques. Magnetic resonance imaging (MRI) is one method that may bridge the gap between invasive techniques used in animal models and cognitive and surface recording assessments used in humans. A recent study indicated maternal iron status and cord blood ferritin measures related to markers of infant brain development, which were assessed using diffusion tensor imaging [20]. While this finding is informative, hematological indices of iron status often do not predict brain iron content, as the brain tends to deplete prior to blood tissue [5]. Thus, a non-invasive assessment of brain iron is needed to quantify iron levels throughout development, thereby illuminating differences that may not be predicted by hematological indices. Quantitative susceptibility mapping (QSM) is an MRI method that has been used to quantify altered iron status in clinical cases of restless leg syndrome [21] and β-thalassemia [22]; thus, it is possible that these methods may be able to sensitively detect altered brain iron status in cases of dietary iron deficiency. In fact, a recent neuroimaging study that used QSM in children found that brain iron content in the caudate nucleus related to performance on spatial intelligence quotient tests [23], thereby offering insight into brain regions that may underlie cognitive differences due to iron status. Moreover, neuroimaging allows for assessment of structural brain development such as myelination, and grey matter and white matter tissue distributions, and may prove useful in sensitively characterizing structural differences later in life.

We previously reported that early-life iron deficiency resulted in decreased relative brain volumes of specific regions and region-specific reductions in diffusion tensor measures, which persisted in developing pig brains even after a subsequent period of dietary iron repletion [24]. Interestingly, these differences in microstructural brain development were present at postnatal day (PND) 61 despite a lack of difference in absolute brain volume between iron-deficient (ID) and control (CONT) pigs at that time-point. These findings suggest comprehensively assessing the effects of iron deficiency is necessary to elucidate region-specific, rather than whole brain, implications of altered nutrient status. To further expand upon our previous findings, herein we analyzed differences between early-life ID and control CONT pigs in concentrations of brain grey matter, white matter, and measures of white matter tract development at PND 61. Due to the highly dynamic nature of the developing brain and the influence of iron on myelin and neuron morphology, we hypothesized that grey and white matter tissue concentrations would be altered by early life iron status and remain evident at PND 61. Thus, the aim of this study was to identify specific regions of the brain that remained structurally different in ID pigs after a subsequent period of iron repletion Additionally we used, a non-invasive neuroimaging technique, QSM, to characterize changes in brain iron content. Using this method, we hypothesized that piglets provided an ID diet early in life would exhibit decreased iron content in the brain, as measured through QSM.

## 2. Materials and Methods

### 2.1. Animal Care and Housing

Twenty-eight, naturally-farrowed, intact male pigs were obtained from Carthage Veterinary Services and transferred to the University of Illinois Piglet Nutrition and Cognition Laboratory (PNCL) at PND 2. Per standard agricultural protocol, pigs were provided an intramuscular injection of a prophylactic antibiotic (0.1 mL of ceftiofur crystalline free acid (Exceed, Zoetis, Parsippany, NJ, USA)) within 24 h of birth. Contrary to typical agricultural procedures, pigs on this study were not administered an intramuscular injection of iron dextran because iron was the nutrient being manipulated as per the experimental design. Recent pig studies observed hippocampal transcriptome changes [25] and possible effects of iron overload [26] after a bolus administration of iron dextran in the first few days of life, which further justifies our decision to not provide iron dextran to any pigs. Upon arrival to PNCL on PND 2, pigs were stratified into one of two experimental diets, described below. Pigs were randomly allocated into treatment groups to account for initial bodyweight and maternal genetics, with no bodyweight or iron status differences between the groups at the start of the study. Pigs were provided experimental milk replacer diets from PND 2 until PND 32 (phase 1), at which point both treatment groups were weaned onto a series of nutritionally-adequate diets from PND 33 until PND 61 (phase 2). A detailed description of phase 1 and phase 2 rearing environments has been previously described [24]. All animal and experimental procedures were in accordance with the National Research Council Guide for the Care and Use of Laboratory Animals and approved by the University of Illinois at Urbana-Champaign Institutional Animal Care and Use Committee. Approval for this research project was confirmed on 3 March 2015 and is identified as IACUC 15034 at the University of Illinois Urbana-Champaign. 

### 2.2. Dietary Treatments

For phase 1 of this study, pigs (*N* = 28; *n* = 14 per diet) were provided one of two dietary treatments with varying iron content. The CONT diet was formulated to meet all of the nutrient requirements of the growing pig and was formulated to contain 117.5 mg Fe/kg milk replacer powder. The ID diet was similar to the CONT diet, however iron was only formulated to be supplemented at 7.8 mg Fe/kg milk replacer powder. Additionally, both diets were formulated to contain arachidonic acid (ARA) (2.08 g ARA/kg milk replacer powder) and docosahexanoeic acid (DHA) (1.04 g DHA/kg milk replacer powder). Milk replacer was reconstituted fresh daily with 200 g of milk replacer powder per 800 g water. Thus, formulated iron concentrations in reconstituted pig milk replacers were: CONT, 23.5 mg Fe/L milk replacer, and ID, 1.56 mg Fe/L milk replacer. All pigs were provided ad libitum access to liquid diets from PND 2 until PND 32. 

For phase 2 of this study, all pigs (*N* = 20, *n* = 10/phase 1 diet) were weaned onto the same series of age-appropriate, nutritionally-adequate solid diets, regardless of their phase 1 dietary iron status. Pigs were provided ad libitum access to water and standard complex diets (major ingredients including corn, whey, and soybean meal) and standard agricultural feeding practices were followed by sequentially switching from stage 1 diets, to stage 2 diets, to stage 3 diets, on PND 33, 41, and 50 respectively. During this phase of the study, all diets were formulated to meet all nutrient requirements of the growing pig [27], including iron. No zinc oxide, copper sulfate, or in-feed antibiotics were included in any diets. 

### 2.3. Magnetic Resonance Imaging

All pigs remaining in each phase underwent MRI procedures on PND 32 or 33 for phase 1 and again at PND 61 or 62 for phase 2, at the Beckman Institute for Advanced Science and Technology Biomedical Imaging Center. For phase 1, 28 pigs (*n* = 14 per diet) were subjected to neuroimaging procedures and for phase 2, 20 pigs (*n* = 10 per phase 1 diet) were subjected to neuroimaging procedures. Imaging procedures were performed using a Siemens MAGNETOM Trio 3T scanner (Siemens, Erlangen, Germany), with a custom pig-specific 8-channel head coil at PND 32 and a human 8-channel head coil at PND 61. Upon arrival to the imaging facility, anesthesia was induced using an intramuscular injection of telazol: ketamine: xylazine solution [50.0 mg tiletamine plus 50.0 mg of zolazepam reconstituted with 2.50 mL ketamine (100 g/L) and 2.50 mL xylazine (100 g/L); Fort Dodge Animal Health] administered at 0.03 mL/kg body weight, and maintained with inhalation of isoflurane (98% O_2_, 2% isoflurane). Pigs were immobilized during all MRI procedures. Visual observation of each pig’s well-being, as well as observations of heart rate, PO_2_ and percent of isoflurane were recorded every 5 min. during the procedure. Total scan time for each pig was approximately 60 min. Upon completion of the scan, pig respiration and heart rate were monitored every 15 min until complete recovery from anesthesia. Imaging techniques are briefly described below. 

#### 2.3.1. Structural MRI Acquisition & Analysis

A T_1_-weighted magnetization-prepared rapid gradient echo (MPRAGE) sequence was used to obtain anatomic images of the pig brain, with a 0.7 mm isotropic voxel size. The following specific parameters were used for the MPRAGE sequence: repetition time (TR) = 1900 ms; echo time (TE) = 2.49 ms; inversion time (TI) = 900 ms; 224 slices; field of view (FOV) = 180 × 180 mm^2^; flip angle = 9°. Pig brains were manually extracted as previously described [28]. All toolboxes described herein were available in SPM12 (Wellcome Department of Clinical Neurology, London, UK) and Matlab R2015a was used for data processing. Once extracted, the ‘Coregister: Estimate & Reslice’ toolbox was used to coregister individual brains to the Pig MRI Atlas [29]. Next, the ‘Old Normalize: Estimate & Reslice’ toolbox was used to transform individual pig brains into atlas space. The following parameters in Old Normalize were used for pig specific data processing: template image (Pig MRI Atlas), bounding box (−30.1 −35 −28/30.1 44.8 31.5), voxel size (0.7). The “Segment” function of SPM12 and pig-specific prior probability tissue maps were then used to segment the brains into grey matter and white matter. Voxel-based morphometry (VBM) analysis was performed to assess grey and white matter tissue concentrations using SPM12 software. The Diffeomorphic Anatomical Registration using Exponentiated Lie Algebra (DARTEL) toolbox was used with pig-specific specifications that included changing the bounding box of −30.1 to 30.1, −35 to 44.8, −28 to 31.5; and a voxel size of 0.7 mm^3^. After the nonlinear transformation of the data in the DARTEL procedure, flow fields were created and converted to warp files. The warp files generated were then applied to the subject’s grey and white matter. The modulated data were smoothed with a 4 mm full-width half maximum, and were subjected to VBM procedures using the SPM12 toolbox. 

#### 2.3.2. Quantitative Susceptibility Mapping

Brain tissue susceptibility was obtained to access the iron content change due to iron-deficiency. To this end, whole brain ^1^H magnetic resonance spectroscopic imaging (MRSI) data without water suppression were acquired using the recently proposed spectroscopic imaging by exploiting spatiospectral correlation SPICE-based acquisition [30,31]. Without water suppression, QSM of brain tissues can be extracted from the phase information encoded in the water spectroscopic signals [31,32]. The detailed acquisition parameters are as follows: TR/TE = 310/4 ms, FOV = 200 × 160 × 64 mm^3^, matrix size = 100 × 120 × 26, flip angle = 37°, readout bandwidth = 167 kHz, number of echoes acquired each TR = 200, and echo spacing = 850 ms. Susceptibility maps were extracted from the water spectroscopic data using the following algorithm (see Peng et al., 2017 [31] for more details): (1) total B_0_ field inhomogeneity maps were estimated by using a voxel-by-voxel least-squares fitting of the multi-echo data; (2) the tissue susceptibility induced field inhomogeneity was extracted from the total field by solving the Laplacian boundary value problem [33]; (3) susceptibility maps were then determined by solving the tissue field to susceptibility dipole inversion [32]; and (4) regional susceptibility values were obtained by averaging the susceptibility values in different regions of interest ROIs, using the University of Illinois Piglet Brain Atlas [29] (http://pigmri.illinois.edu/). 

#### 2.3.3. Tract-Based Spatial Statistics

Diffusion tensor imaging was used to assess white matter maturation and axonal tract integrity using a *b*-value = 1000 s/mm^2^ across 30 directions and a 2 mm isotropic voxel. Diffusion-weighted echoplanar imaging EPI images were assessed in FMRIB Software Library (FSL) for fractional anisotropy (FA), mean diffusivity (MD), axial diffusivity (AD), and radial diffusivity (RD) using methods previously described [28]. The FSL 5.0 toolbox was used for tract-based spatial statistics (TBSS) assessment of FA data [34,35]. Fractional anisotropy images, previously generated from diffusion data, were manually extracted, and all FA data from individual subjects were aligned using the FSL nonlinear registration tool FNIRT. Upon alignment, the study-specific mean FA image was created and a mean FA skeleton representing the center of all common tracts was established. A threshold of 0.2 was determined to be sensitive for mean FA tract delineation. Once the study-specific mean FA skeleton was created, each subjects’ aligned FA data were projected onto the mean FA skeleton and the resulting voxel-wise cross-subject data were used for statistical analyses [34,35]. For all TBSS analyses involving registration to an atlas, the University of Illinois Piglet Brain Atlas (http://pigmri.illinois.edu/) was used in place of human brain templates [29]. 

### 2.4. Statistical Analysis

All researchers involved in this study (i.e., those performing daily procedures, data collection, and data analysis steps) remained blinded to dietary treatment identity until final data analyses had been completed. Data were analyzed by using the MIXED procedure of SAS 9.4 (SAS Institute, Cary, NC, USA). Quantitative susceptibility measures were analyzed from data generated at PND 32 and 61, thus all data were analyzed using a 2-way repeated measures analysis of variance (ANOVA) (i.e., dietary iron status with postnatal day at time of neuroimaging acquisition as the repeated measure). Interactive effects were defined as an interaction between diet (CONT vs. ID) and MRI day (PND 32 vs. 61). The number of animals per treatment group was based on a power analysis using the variability estimates from previous studies to detect differences with sufficient power of 80% and at a significance of 0.05. Data were analyzed for outliers (defined as having a studentized residual with an absolute value greater than 3) and outliers were removed prior to statistical analysis. Significance was accepted at *p* ≤ 0.05. Data are presented as least-squares means with pooled standard errors of the mean (SEM). 

#### 2.4.1. Voxel-Based Morphometry Statistics

Voxel-based morphometry analyses were performed on data acquired at PND 61. As such, two-sample permutation *t*-tests were performed on a voxel-by-voxel basis for grey and white matter volume differences between animals on the early-life CONT and ID diets, with an uncorrected *p <* 0.001. An additional threshold criterion of at least 20 edge-connected voxels was used. 

#### 2.4.2. Tract-Based Spatial Statistics

For TBSS analysis, only data acquired at PND 61 was analyzed. A nonparametric permutation inference function called ‘randomise’ was used within the FSL toolbox and run as a two-sample *t*-test with 500 permutations to compare the effects of early-life CONT and ID diets. Multiple comparisons were also accounted for within the randomize function. The resulting statistical analysis was then presented as heat maps indicating brain areas where FA values differed between dietary treatments.

## 3. Results

### 3.1. Quantitative Susceptibility Mapping

**Figure 1:**
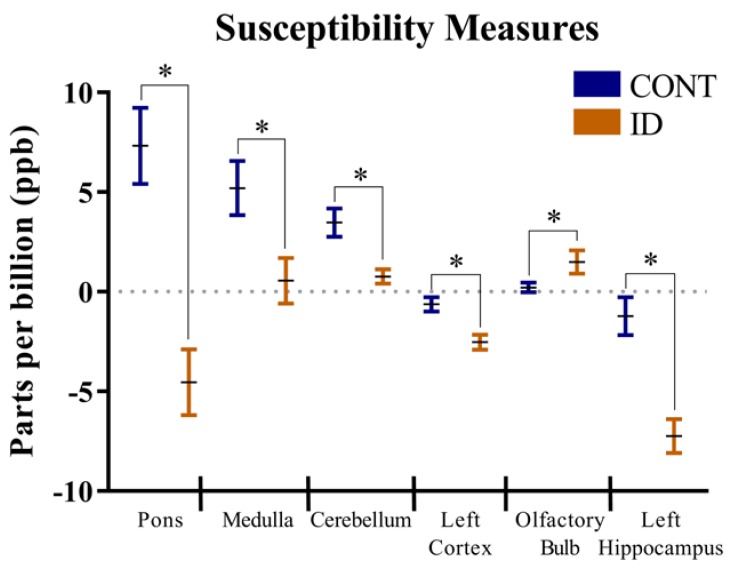
Measures of average iron content in brain regions were influenced by dietary iron status, regardless of imaging time-point. Because there was no significant interaction between diet and magnetic resonance imaging (MRI) day, this figure only shows the significant main effects of diet, regardless of time. Reduced iron content in the pons (*p* < 0.001), medulla (*p* = 0.018), cerebellum (*p* = 0.005), left cortex (*p* = 0.004), and left hippocampus (*p* < 0.001) was observed in ID pigs compared with CONT pigs. Iron content of the olfactory bulb was increased (*p* = 0.043) in ID pigs compared with CONT pigs. Note that as iron content increases, quantitative susceptibility measures values change from diamagnetic (negative values) to paramagnetic (positive values). Abbreviations: control (CONT); iron deficient (ID). * Main effect of early-life dietary iron status, differs by *p* < 0.05.

Interactive effects of diet and MRI day were not observed for region-specific QSM measures. A main effect of dietary treatment (i.e., independent of MRI day) (*p* < 0.05) was observed in the pons, medulla, cerebellum, left cortex, olfactory bulb, and left hippocampus, Figure 1 and Table 1. Regardless of imaging time-point, pigs receiving the ID diet exhibited lower QSM values in the pons (*p* < 0.001), medulla (*p* = 0.02), cerebellum (*p* < 0.01), left cortex (*p* < 0.01), and left hippocampus (*p* < 0.001) when compared with CONT pigs. Conversely, pigs on the ID diet exhibited higher (*p* = 0.04) QSM values in the olfactory bulb when compared with CONT pigs, regardless of imaging time-point. 

A main effect of MRI day (i.e., independent of early-life iron status) (*p* < 0.05) was observed for corpus callosum, hypothalamus, and right cortex. The QSM measures in the corpus callosum (*p* = 0.03) increased from 0.26 ± 1.36 ppb on PND 32 to 4.89 ± 1.36 ppb at PND 61. In the right cortex, QSM measures increased (*p* = 0.03) from −1.83 ± 0.42 ppb on PND 32 to −0.21 ± 0.42 ppb at PND 61. Conversely, the QSM measures in hypothalamus decreased (*p* = 0.04) from −3.65 ± 1.70 ppb on PND 32 to −8.62 ± 1.70 ppb on PND 61. Notably, as iron content increases, QSM measures change from negative (i.e., indicating diamagnetic tissue properties) to positive (i.e., indicating paramagnetic tissue properties). Note, no interactive effects were observed, but means for both dietary treatments at both imaging time points are presented in Table 1. Accordingly, a main effect of diet can be determined by averaging the measures within dietary treatment group for each brain region and a main effect of time can be determined by averaging the two treatment groups at each imaging time point. 

### 3.2. Voxel-Based Morphometry

**Figure 2:**
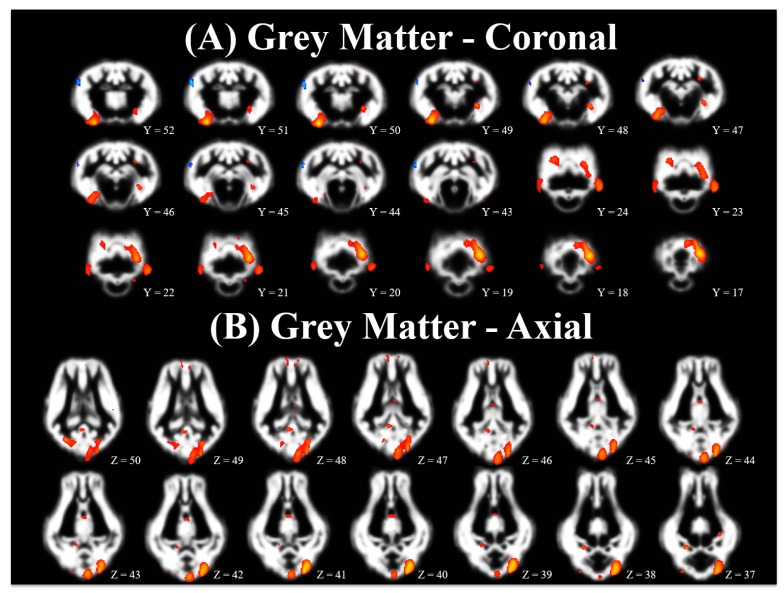
Pictured here is a population-averaged pig brain, with a statistical heat map indicating differences in grey matter between dietary treatment groups. The range of red-to-yellow indicates the degree of statistical difference from significant pseudo-*t* values of 3.80 to 7.35, respectively, in voxels where CONT pigs exhibited increased grey matter concentrations compared with ID pigs (i.e., CONT grey matter > ID grey matter). Clusters that range from dark-to-light blue indicate increasing significance from significant pseudo-*t* values of 4.30 to 5.55, respectively, in voxels where ID pigs exhibit increased grey matter compared with CONT pigs (i.e., ID grey matter > CONT grey matter). (**A**) Brain images in coronal orientation and (**B**) Brain images in axial orientation. Abbreviations: control (CONT); iron deficient (ID).

**Figure 3:**
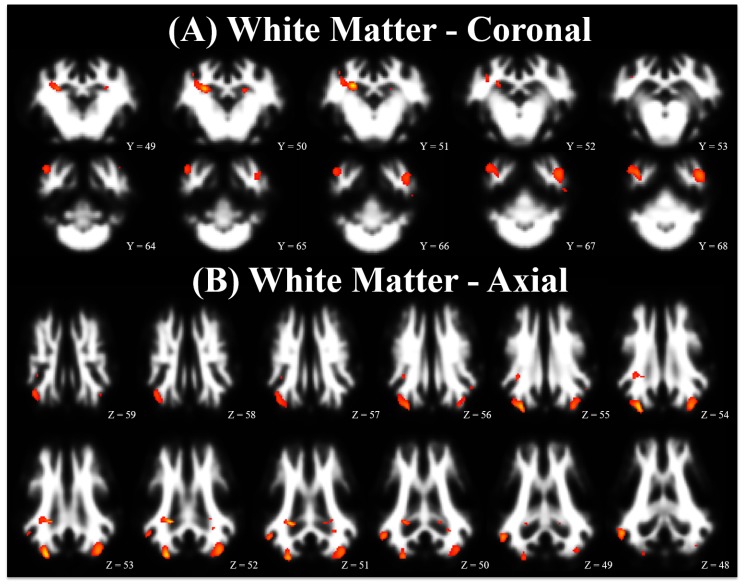
Pictured here is a population-averaged pig brain, with a statistical heat map indicating differences in white matter between dietary treatment groups. The range of red-to-yellow indicates the degree of statistical difference from significant pseudo-*t* values of 4.00 to 6.40, respectively, in voxels where CONT pigs exhibited increased white matter concentrations compared with ID pigs (i.e., CONT white matter > ID white matter). Notably, no differences were observed where ID pigs exhibited increased white matter concentrations compared with CONT pigs. (**A**) Brain images in coronal orientation and (**B**) Brain images in axial orientation. Abbreviations: control (CONT); iron deficient (ID).

Voxel-based morphometry is an analytical technique used to compare grey or white matter tissue distribution between two treatment groups. Accordingly, each treatment group may have more or less grey matter or white matter, resulting in clusters of voxels that indicate differences in tissue composition. Analysis of grey and white matter tissue segmentations from the PND 61 imaging time-point indicated region-specific differences (*p* < 0.001) between CONT and ID pigs, Table 2. A comparison of grey matter voxels where CONT pigs exhibited more (*p* < 0.001) grey matter voxels than ID pigs indicated differences in the left cortex, right cortex, cerebellum, hippocampus, thalamus, and caudate (CONT > ID), Figure 2 (red comparisons). The opposite comparison, in which ID pigs exhibited increased grey matter (*p* < 0.001) compared with CONT pigs (ID > CONT) indicated small grey matter clusters, in each of the left and right cortex, Figure 2 (blue comparisons). Comparison of white matter voxels where CONT pigs exhibited more (*p* < 0.001) white matter voxels than ID pigs indicated differences in the internal capsule, left cortex, right cortex, left hippocampus, and right hippocampus (CONT > ID), Figure 3 (red comparisons) and Table 2. There were no instances in which ID pigs exhibited more white matter compared with CONT pigs (ID > CONT). Table 2 lists clusters of edge-connected voxels where a statistical difference between the two dietary treatments was observed. The anatomical regions described were determined from *X, Y, Z* coordinates that corresponded to peak maximum differences within the clusters, thus it is possible to have multiple peaks per cluster. 

### 3.3. Tract-Based Spatial Statistics

**Figure 4:**
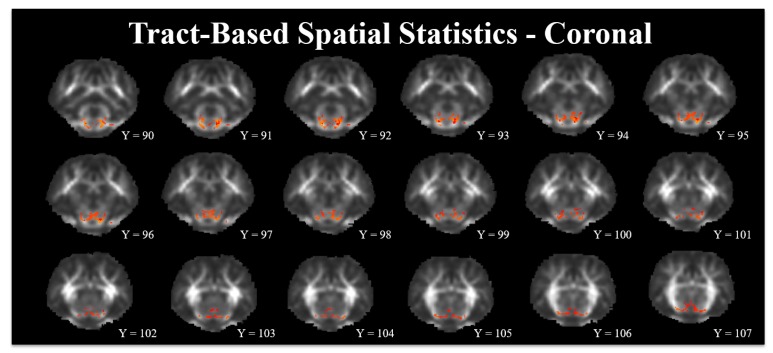
Pictured here is a population-averaged pig brain, with a statistical heat map indicating differences in white matter tract development between dietary treatment groups. Fractional anisotropy (FA) differences along predetermined white matter tracts where CONT pigs exhibited higher (*p* < 0.05) FA values compared with ID pigs. Representative slices were chosen to highlight areas in which FA values in CONT pigs were higher than in ID pigs. The range of red-to-yellow indicates degree of statistical difference from *p* = 0.05 to *p* = 0.001, respectively. Brain images in coronal orientation. Abbreviations: control (CONT); fractional anisotropy (FA); iron deficient (ID).

Tract-based spatial statistics allows assessment of differences due to diet in FA values along predetermined white matter tracts from data acquired at the PND 61 time-point. Analysis of FA values indicated subcortical areas in which CONT pigs exhibited greater (*p* < 0.05) FA values compared with ID pigs, Figure 4. Upon visual inspection, the greatest FA value differences appear to be in the white matter tracts located in the caudate and thalamus. Analysis of the opposite comparison, where FA values were greater in ID pigs compared with CONT pigs, revealed no differences (*p* > 0.05). 

## 4. Discussion

Iron is pivotal for proper brain development, and alterations in both prenatal and postnatal iron status severely influence brain structure and function [4,6,16,36]. Moreover, brain development is a dynamic and heterogeneous process, thus the effects of iron deficiency are highly dependent on the timing and severity of the altered iron status [6]. In our study, pigs were provided either an ID or CONT diet from PND 2 until PND 32, at which point all pigs were switched to iron-replete diets until PND 61. The aim of this study was to determine what microstructural differences persisted in the brain at PND 61, despite all pigs being provided iron-replete diets from PND 33 to PND 61. In doing so, our study was designed to mimic postnatal dietary iron deficiency in human infants from birth up until four to six months of age when iron-fortified foods tend to be introduced into the diet. We previously published results indicating that total brain volumes are different between ID and CONT pigs at PND 32 but are not different at PND 61 [24]. Despite the observed compensatory brain volume growth during the period of dietary iron repletion, results from our analyses indicated tissue microstructural differences remained at PND 61. Therefore, to further elucidate how dietary iron status influenced brain development, we used voxel-based morphometry and tract-based spatial statistics to provide visual characterizations of neurodevelopment at PND 61. Herein we provide evidence that structural differences in grey and white matter are present in specific regions of the brain, even after 30 days of iron repletion. We also utilized quantitative susceptibility mapping to non-invasively characterize differences in brain iron content due to early-life dietary iron deficiency. These results are poised to have clinical relevance as we show neuroimaging can sensitively quantify differences in brain iron content due to dietary iron status. 

### 4.1. Quantitative Susceptibility Measures

Iron is essential throughout early-life brain development to facilitate proper myelination [15], neurotransmitter synthesis [16,17], and neuron morphology [18,19]. Accordingly, the concentration of iron varies by brain region [37,38] and its presence is critical to ensure proper development throughout the brain. To date, many animal studies have focused on quantification of brain iron using invasive techniques to analyze samples of brain tissue [14,26,39]. Importantly, assessment of iron status through blood biomarkers is not always a reliable predictor of brain iron content. As evidence of this, a recent study where pigs were provided with ID diets early in life followed by iron replete diets indicated hematocrit and hemoglobin measures were not different between CONT and ID pigs at 12 weeks of age, but hippocampal iron content remained lower in ID pigs [26]. Provided the ethical limitations of collecting brain samples from human infants and the unreliable relationship between blood and brain iron concentrations, there is a need for a non-invasive assessment of brain iron content early in life. Quantitative susceptibility mapping is a non-invasive neuroimaging technique which can be used to sensitively characterize brain iron content [32]. This technique quantifies magnetic properties of tissue, and as iron is deposited into tissue, the tissue becomes increasingly paramagnetic. Thus, as development occurs and iron is accreted, it is expected that QSM values will increase (indicating stronger paramagnetic tissue properties). A previous report, where QSM methods were used in children, indicates that increased brain iron concentrations in the caudate related to increased spatial IQ [23]. Accordingly, this method may prove to be a sensitive non-invasive biomarker for quantifying brain iron content, and may help to explain differences in brain structural and functional development. To our knowledge, there do not appear to be any studies using QSM to determine the iron status of infant brains, however this method has been shown to sensitively characterize altered iron content in clinical cases of restless leg syndrome [21] and β-thalassemia [22]. 

In the present analysis, we used QSM to assess whether differences in brain iron could be detected non-invasively after a period of early-life iron deficiency. Our results indicate that pigs provided an early-life ID diet exhibited decreased brain iron concentrations in the cerebellum, pons, medulla, left cortex, and right cortex compared with CONT pigs at PND 32 and after receiving iron replete diets (i.e., at PND 61). Interestingly, ID pigs exhibited increased iron content in the olfactory bulb compared with CONT pigs. Previous analysis of brain volumes and diffusion tensor measures in this group of pigs indicated decreased relative brain volumes in the left hippocampus and the cerebellum of ID pigs [24]. Iron is needed for growth and expansion of neurons as well as dendrite morphogenesis in rodents [40] and pigs [41]. Thus, our QSM data may suggest a mechanism whereby decreased brain iron content attenuated neuron growth in the left hippocampus and cerebellum, thereby resulting in reduced relative volumes of these regions. The suggestion that iron is necessary for neuron growth is also supported by our findings of increased brain iron content in the olfactory bulbs of ID pigs and our previous report of increased relative volumes of the olfactory bulbs in ID pigs [24]. It remains to be elucidated why the olfactory bulb exhibits opposite growth trends during an ID state, however this finding of increased brain iron content in the olfactory bulb adds to evidence that this is likely not a spurious finding. We previously reported decreased FA values in the left cortex and the cerebellum of ID pigs. Fractional anisotropy is often used as a non-invasive marker of myelination and fiber coherence in the brain and increases throughout development [42,43]. It is also known that iron is an essential co-factor in myelinating events [15,44,45]. Thus, the decreased iron concentrations in the cerebellum and left cortex further support our hypothesis of decreased myelination in these two brain regions in young pigs. 

Interestingly, a study of brain iron content in adults ranging from 22 to 78 years of age indicated increased iron content in left hemisphere brain regions compared with right hemisphere regions [46]. Xu and colleagues [46] suggest that this hemispheric difference in iron content may be due to lateralization of motor control, which is largely controlled by the dopaminergic system and is dependent on iron for neurotransmitter synthesis. Although handedness in our pigs was not assessed, our data of decreased iron in the left hippocampus and left cortex in ID pigs compared with CONT pigs may suggest an apparent hemispheric sensitivity of brain iron accumulation due to dietary iron status. In a rodent model of chronic early-life iron deficiency followed by iron repletion into adulthood, concentrations of brain iron restored to levels not different than in CONT animals in all regions except the thalamus [17]. Results from our study assessed brain iron at PND 32 and 61 and only a main effect of dietary treatment was noted for the brain regions discussed. Thus, future research should seek to non-invasively characterize brain iron content after a longer period of repletion to identify if iron content in ID pig brains is able to recover to levels observed in CONT pigs. In doing so, researchers will be able to better define the extent of dietary iron adequacy that is necessary to restore brain iron content back to baseline levels within an individual. It should be noted, however, that Felt and colleagues [17] observed altered monoamine metabolism and behavior in adult rodents that were previously ID, indicating that restoring brain iron to normal levels may not be sufficient to counteract the alterations in brain development due to early life iron deficiency. Our QSM findings of altered brain iron content stand to have clinical relevance, as we were able to use a non-invasive neuroimaging technique to sensitively quantify differences in brain iron content between ID and CONT pigs. Knowing that brain iron tends to decrease prior to hematological decreases in iron [5], neuroimaging of at-risk infants may help to identify and correct alterations in brain iron before overt signs of clinical iron deficiency are observed. 

It is also worth mentioning that three brain regions indicated a change of iron content over time that was independent of dietary treatment. Notably, we observed an increase in QSM values in the corpus callosum and right cortex from PND 31 to PND 62, whereas a decrease in values was observed in the hypothalamus. Because these findings did not indicate sensitivity to dietary treatment, it suggests that these regions might be more resilient or less affected by alterations in dietary iron status when accumulating iron-containing compounds. We previously reported no differences due to dietary iron status in diffusion tensor fractional anisotropy, a measure indicating myelin and fiber tract development, in the corpus callosum [24]. Despite the lack of dietary effect in the corpus callosum FA values [24], we did observe an increase in corpus callosum FA values over time, which is consistent with the QSM values we report herein. Notably, our analysis of FA values in the right cortex did indicate a dietary effect [24], whereas our QSM values did not exhibit an effect of diet. This might suggest that the presence of iron-containing compounds is not solely responsible for changes in brain structure. As this is one of the first studies to assess QSM over time in early development, it is unclear if the decrease in hypothalamus values is of physiological relevance. Thus, future work should seek to better characterize changes in QSM across multiple time points to elucidate the trajectory of QSM changes from birth until adulthood. Doing so in the context of a longer period of iron deficiency might illuminate brain regions that are susceptible to alterations in QSM at different time points throughout the developmental process. 

### 4.2. Voxel-Based Morphometry

We previously reported a reduction of approximately 10% in total brain volume at PND 32 in ID pigs compared with CONT pigs. However, after a period of dietary iron repletion from PND 33 to 61, brain volumes were not statistically different between early life ID and CONT pigs [24]. Despite this finding of similar brain volume, we observed microstructural differences in diffusion tensor measures between the two groups at PND 61, indicating that dietary iron repletion did not recover all aspects of brain development. Therefore, to further explore these microstructural differences we used voxel-based morphometry to visually assess differences in grey and white matter tissue concentrations that remained present at PND 61. Assessment of grey matter tissue differences between CONT and ID pigs indicated more voxels in which CONT pigs had greater concentrations of grey matter compared with ID pigs. Interestingly, these clusters of increased grey matter in CONT pigs were located in the left cortex, right cortex, cerebellum, thalamus, caudate, and right hippocampus. A previous study of iron deficiency in 30-day-old pigs indicated differences in cortical grey matter in CONT pigs compared with ID pigs [47]. In our study and the study by Leyshon and colleagues, the most abundant differences in grey matter appeared to be localized to cortical brain regions. However, the differences that were observed by Leyshon and colleagues between CONT and ID pigs at 30 d of age contained more voxels and larger clusters than what we observed in our pigs after a period of iron repletion on PND 61. Thus, it is possible that a period of iron repletion may be able to compensate for some alterations in tissue grey matter, yet it is clear that structural differences are present after 30 days of iron repletion. 

In a rodent model of perinatal iron deficiency, ID rodents exhibited decreased branching complexity of cortical apical and basal dendrites, but no difference in dendrite length when compared with CONT rodents [19]. Pigs that were provided ID diets from birth until 4 weeks of life and iron replete diets from 4 until 12 weeks of life exhibited no difference in brain-derived neurotrophic factor (BDNF) expression in the prefrontal cortex [48]. In pigs, cortical brain tissue is just reaching its maximal growth rate at 4 weeks of age [49], corresponding to the age at which our study and the study by Nelissen and colleagues [48] switched ID pigs to iron replete diets. Thus, the lack of difference in BDNF in the prefrontal cortex of 12-week-old pigs and our observation of similar brain volumes at 8 weeks of age between ID and CONT pigs may suggest neuron outgrowth was not drastically influenced by early-life iron deficiency. However, the persistent differences in localized grey matter concentrations in the cortex may suggest altered neuron morphology, thus corroborating the findings by Greminger and colleagues [19]. The observed reductions in right hippocampal grey matter in ID pigs may also be a result of altered dendritic arborization, as this has previously been observed in ID hippocampal cell cultures from rodents [18]. These findings may suggest a non-invasive technique for characterizing altered cortical neuronal complexity in pigs that were ID early in life. 

When assessing white matter, CONT pigs exhibited localized increases in both hippocampi, both cortices, the cerebellum, and the internal capsule when compared with ID pigs. In contrast, assessment of regions in which ID pigs exhibited increased white matter compared with CONT pigs yielded no significant results. This finding substantiates previous findings in 30-day-old pigs in which ID pigs did not have any voxels containing more white matter when compared with CONT pigs [47]. Together, these findings indicate that once detriments in white matter maturation have occurred early in life, recovery may not be possible, even after a subsequent period of dietary iron repletion. This observation of decreased white matter also aligns with our previous results of decreased FA values in the whole brain, cortex, cerebellum, and internal capsule due to early-life iron deficiency in pigs [24]. Previous research indicates reduced cerebellar myelination in rodents [45], thus suggesting that our observed reductions in cerebellar white matter may be due to reduced myelination. A previous study of 30-day-old ID pigs also indicated reduced white matter concentrations in the internal capsule [47]. It is known that the internal capsule and cerebellum are critical for coordinating motor skills and previous research in ID and ID anemic infants indicates delayed motor development [8], thus our findings of reduced white matter concentrations in these regions may indicate reductions in myelin development. While our study did not assess motor coordination or gait analysis in young pigs, future work should seek to assess the effect of early life iron on these measures. Provided there remain differences in both grey and white matter after a 30 day period of dietary iron repletion, future studies should seek to characterize if there is a period of iron repletion that is capable of rendering CONT and ID brains structurally similar.

### 4.3. Tract-Based Spatial Statistics

Tract-based spatial statistics allows for comparison of diffusion values along predetermined white matter tracts in the brain. Our previous diffusion tensor analysis indicated reductions in whole brain FA values in ID pigs, as well as FA reductions in the caudate, cerebellum, and internal capsule [24]. These previous observations were averaged over all white matter found in a defined brain region and were not specific to white matter tracts. Provided our previous results indicated differences in diffusion values that persisted at PND 61, we used TBSS to visualize white matter tracts where early-life ID pigs exhibited decreased FA values relative to CONT pigs. Assessment of white matter tracts in which CONT pigs exhibited voxels with higher FA values compared with ID pigs indicated differences located exclusively in subcortical brain regions. From our analysis, the regions in which FA values were greater in CONT pigs along white matter tracts appear to be localized to the internal capsule, thalamus, and hypothalamus. Importantly, there were no voxels along white matter tracts in which ID pigs exhibited increased FA values compared with CONT pigs, thereby suggesting that iron deficiency only causes detriments in white matter integrity and does not support white matter maturation. As described above, decreased white matter was observed in the internal capsule of ID pigs compared with CONT pigs. Thus, our VBM findings and the differences in TBSS in the internal capsule may suggest decreased myelin content of this brain region. Moreover, ID and ID anemic infants are known to have delayed motor development [8], and both the internal capsule and thalamus contain motor projections within the brain [50,51]. Previous research in rodents indicates the thalamus is susceptible to dietary iron deficiency, resulting in alterations in monoamine metabolism [4,16,17]. Additionally, increased thalamic axial, radial, and mean diffusivity values were observed in ID pigs at PND 29, suggesting altered myelination in this region [47]. Thus, our findings of altered white matter tracts in these two regions may suggest decreased internal capsule and thalamic myelination, thereby affirming a mechanism through which motor development is delayed in ID infants. It is interesting to note that these differences persist despite a subsequent period of iron repletion in pigs, thus indicating either the period for iron repletion was not long enough or it was applied too late in development to allow for recovery of microstructural changes due to early-life iron deficiency. Future research should seek to quantify myelin content in these brain regions at different time-points during and after iron deficiency to more definitively link iron status and myelination. 

### 4.4. Limitations

While the results of this study are novel, we cannot definitively conclude that iron repletion after a period of early-life iron deficiency is incapable of compensating for developmental differences. Future research should seek to quantify different periods of dietary iron repletion as well as the timing of first providing an iron replete diet. In doing so, researchers will be able to better quantify critical windows during which iron influences particular aspects of brain development. Provided the results of our study, we suggest that early-life iron deficiency through PND 32 in the pig greatly alters myelinating events and may influence morphology of grey matter, but it does not appear to influence overall brain growth. 

## 5. Conclusions

We previously showed that iron deficiency alters whole brain volumes at PND 32 but iron repletion was able to correct for observed differences by PND 61. Here we have shown that despite similar brain volumes, differences in grey matter and white matter, as well as decreased white matter tract integrity, remain at PND 61. Thus, while gross brain morphology appeared unaltered, microstructural detriments in brain development persisted in pigs exposed to early-life iron deficiency followed by a period of iron repletion. Our results also indicate region-specific reductions in brain iron concentrations of ID pigs, regardless of imaging time-point. Notably, characterization of altered brain iron and grey and white matter tissue concentrations were observed through non-invasive techniques, thereby providing clinically relevant methods to assess similar results in human infants. Thus, it is possible that neuroimaging may be used to comprehensively characterize altered brain iron status in infants at risk of iron deficiency, prior to the presence of overt symptoms of iron deficiency or iron deficiency anemia.

## Figures and Tables

**Table 1:** Quantitative susceptibility measures indicating iron concentrations (ppb) in defined brain regions of pigs differing in early-life iron status ^1^.

Title	CONT		ID			*p*-Value		
*Region of interest*	PND 32	PND 61	PND 32	PND 61	SEM	Diet	Day	Diet × Day
Caudate	−6.3	−3.4	−2.1	−2.4	1.51	0.103	0.318	0.217
Cerebellum	4.6	1.6	0.9	0.5	0.91	0.005	0.069	0.144
Cerebral Aqueduct	−9.0	−15.8	−13.3	−9.2	3.14	0.723	0.609	0.065
Corpus Callosum	0.6	5.3	−0.1	4.5	2.04	0.676	0.029	0.994
Fourth Ventricle	−1.1	−9.8	−3.6	−7.5	3.59	0.985	0.057	0.428
Hypothalamus	−4.7	−11.0	−2.6	−6.3	2.48	0.161	0.044	0.550
Internal Capsule	−8.6	−8.0	−9.5	−7.5	1.04	0.760	0.303	0.560
Lateral Ventricle	−3.8	−1.3	−4.3	−2.4	1.48	0.542	0.110	0.834
Left Cortex	−0.6	−0.5	−2.2	−2.8	0.60	0.004	0.614	0.470
Left Hippocampus	−0.5	−1.9	−7.6	−6.9	1.53	<0.001	0.811	0.447
Medulla	5.1	5.3	−0.2	1.5	2.09	0.018	0.666	0.729
Midbrain	−6.5	−6.0	−8.8	−7.8	2.15	0.351	0.684	0.867
Olfactory Bulb	0.4	−0.2	1.6	1.5	0.77	0.043	0.601	0.715
Pons	4.7	10.9	−6.5	−2.2	2.83	<0.001	0.064	0.702
Putamen-Globus Pallidus	−6.3	−3.9	−5.1	−4.5	1.22	0.786	0.175	0.392
Right Cortex	−1.0	0.0	−2.7	−0.5	0.72	0.083	0.033	0.383
Right Hippocampus	−3.2	−0.6	−7.0	−4.2	2.29	0.141	0.127	0.979
Thalamus	−7.7	−11.0	−7.0	−7.6	1.88	0.322	0.166	0.319
Third Ventricle	−4.1	−4.8	−1.8	0.9	2.49	0.105	0.663	0.433

^1^ Data presented as least square means and pooled standard errors of the mean (SEM) for each treatment group. Statistical significance of the main effects of early-life dietary treatment (Diet; CONT vs. ID) and postnatal magnetic resonance imaging (MRI) day (Day; PND 32 vs. 61) and the interaction between Diet and Day are presented. Number of pigs per treatment group that were subjected to MRI are as follows: PND 32 (CONT, *n* = 9–11; ID, *n* = 8–10), PND 61 (CONT, *n* = 5–7; ID, *n* = 7–9). In general susceptibility measures increase from negative (diamagnetic) to positive (paramagnetic) as iron accumulates in tissues. Abbreviations: control (CONT), iron deficient (ID), parts per billion (ppb), postnatal day (PND).

**Table 2:** Voxel-based morphometry assessment of grey and white matter at PND 61 comparing pigs from differing early-life iron status ^1^.

			Cluster Level		Peak Level		Local Maxima Coordinates ^3^		
Tissue	Comparison	Anatomic Region ^2^	Number of Voxels	*p*-Value	*p*-Value	Pseudo-*t*	*X*	*Y*	*Z*
Grey	CONT > ID	Right Cortex	2045	<0.001	<0.001	7.31	14.0	0.0	−12.6
		Right Cortex			<0.001	5.32	18.9	−13.3	−8.4
		Cerebellum	2772	<0.001	<0.001	6.92	−10.5	−25.2	0.0
		Cerebellum			<0.001	5.98	−2.8	−27.3	2.8
		Left Cortex			<0.001	5.16	−10.5	−16.8	5.6
		Right Cortex	443	0.004	<0.001	5.98	11.9	−15.4	7.7
		Cerebellum	997	<0.001	<0.001	5.55	−18.9	−18.9	−7.7
		Cerebellum	251	0.023	<0.001	5.54	9.1	−9.8	−2.1
		Right Hippocampus			<0.001	5.12	2.1	−9.1	6.3
		Left Cortex	242	0.025	<0.001	5.27	−14.0	−1.4	−3.5
		Left Cortex	204	0.037	<0.001	5.01	−13.3	−7.0	13.3
		Cerebellum	47	0.291	<0.001	4.83	11.9	−25.9	−2.8
		Thalamus	160	0.061	<0.001	4.36	0.0	10.5	0.7
		Right Cortex	79	0.175	<0.001	4.35	3.5	36.4	4.9
		Right Cortex	72	0.194	<0.001	4.15	13.3	−9.8	11.2
		Left Cortex	24	0.455	0.001	3.97	−5.6	−20.3	11.9
		Left Cortex	42	0.318	0.001	3.97	−2.1	33.6	5.6
		Caudate	21	0.486	0.001	3.81	2.1	18.2	2.8
Grey	CONT <ID	Right Cortex	419	0.005	<0.001	5.51	22.4	0.7	10.5
		Left Cortex	57	0.246	<0.001	4.45	−19.6	4.2	7.0
		Right Cortex	35	0.363	<0.001	4.34	18.2	16.1	12.6
White	CONT > ID	Right Hippocampus	234	0.022	<0.001	6.37	8.4	−2.1	8.4
		Right Cortex	629	0.001	<0.001	6.19	10.5	−16.8	9.1
		Right Cortex			<0.001	4.61	15.4	−9.8	13.3
		Right Cortex	378	0.005	<0.001	5.85	20.3	−7.0	4.2
		Left Cortex	653	0.001	<0.001	5.26	−14.0	−15.4	9.1
		Cerebellum			<0.001	4.26	−18.9	−16.1	0.7
		Internal Capsule	51	0.252	<0.001	4.48	−11.2	−5.6	7.7
		Left Hippocampus	61	0.212	<0.001	4.42	−7.0	−1.4	7.0
		Left Cortex	26	0.415	<0.001	4.07	−18.9	−7.7	11.2
		Internal Capsule	40	0.310	0.001	4.00	−8.4	20.3	3.5
White	CONT < ID	None	-	-	-	-	-	-	-

^1^ Voxel-based morphometry analysis of gray and white matter differences in the CONT and ID pig brains at PND 61. A threshold of *p* <0.001 and minimum cluster size of 20 voxels were used to determine *p*-uncorrected values listed in the table. Abbreviations: control (CONT), iron deficient (ID). ^2^ Brain regions based on visual inspection of the cluster location and cross-referenced with the Piglet Brain Atlas [29]. ^3^ Local maxima coordinates: *X* increases from left (−) to right (+), *Y* increases from posterior (−) to anterior (+), and Z increases from inferior (−) to superior (+).
